# Reactive oxygen species mediate tapetal programmed cell death in tobacco and tomato

**DOI:** 10.1186/s12870-017-1025-3

**Published:** 2017-04-20

**Authors:** Shi-Xia Yu, Qiang-Nan Feng, Hong-Tao Xie, Sha Li, Yan Zhang

**Affiliations:** State Key Laboratory of Crop Biology, College of Life Sciences, Shandong Agricultural University, Tai’an, 271018 China

**Keywords:** Male sterility, Pollen, Anther development, Tapetum, ROS

## Abstract

**Background:**

Hybrid vigor is highly valued in the agricultural industry. Male sterility is an important trait for crop breeding. Pollen development is under strict control of both gametophytic and sporophytic factors, and defects in this process can result in male sterility. Both in the dicot Arabidopsis and in the moncot rice, proper timing of programmed cell death (PCD) in the tapetum ensures pollen development. Dynamic ROS levels have been reported to control tapetal PCD, and thus pollen development, in Arabidopsis and rice. However, it was unclear whether it is evolutionarily conserved, as only those two distantly related species were studied.

**Results:**

Here, we performed histological analyses of anther development of two economically important dicot species, tobacco and tomato. We identified the same ROS amplitude during anther development in these two species and found that dynamic ROS levels correlate with the initiation and progression of tapetal PCD. We further showed that manipulating ROS levels during anther development severely impaired pollen development, resulting in partial male sterility. Finally, real-time quantitative PCR showed that several tobacco and tomato *RBOH*s, encoding NADPH oxidases, are preferentially expressed in anthers.

**Conclusion:**

This study demonstrated evolutionarily conserved ROS amplitude during anther development by examining two commercially important crop species in the Solanaceae. Manipulating ROS amplitude through genetic interference of *RBOH*s therefore may provide a practical way to generate male sterile plants.

**Electronic supplementary material:**

The online version of this article (doi:10.1186/s12870-017-1025-3) contains supplementary material, which is available to authorized users.

## Background

Hybrid vigor is highly valued in agricultural industry, and thus male sterile lines are favored in generating hybrids commercially. In addition, male sterility is a trait critical for biomass production, as plant senescence proceeds much faster in fertile strains than in sterile strains [1, 2]. Therefore, breeding new varieties of male sterile lines is not only important for the seed industry to generate hybrids at low cost but also critical for delaying senescence and increasing plant biomass.

Defective pollen development is a major factor causing male sterility. Pollen development can be affected by tapetal irregularities [3–6], cytoskeletal alterations [7, 8], aberrations in auxin metabolism [9, 10], and altered sugar utilization. Among all the factors controlling pollen development, the tapetum, a layer of sporophytic cells surrounding the pollen sac, plays an essential role [11]. The tapetum undergoes a programmed cell death (PCD) process during pollen development, to provide enzymes to release microspores from tetrads, to mediate microspore development, and to deposit components for the pollen coat [3, 12, 13]. The timing and progression of tapetal PCD is tightly controlled by evolutionarily conserved transcriptional networks [3, 12–17].

In addition to the transcriptional networks regulating tapetal PCD, recently intracellular factors have been identified during this process. Reactive oxygen species (ROS), including hydrogen peroxide and superoxide, affect a large number of proteins through post-translational modifications and ROS also act as critical signaling molecules [18–20]. ROS plays a key role in tapetal PCD both in Arabidopsis and in rice [21–23]. Failure to remove ROS in the tapetum resulted in male sterility, due to precocious tapetal cell death [21–23] whereas reducing ROS levels during anther development by mutation of *RBOH*s, genes encoding NADPH oxidases, delayed tapetal PCD [22]. In both situations, pollen development was severely impaired [21–23].

Although the essential role of dynamic ROS levels for tapetal PCD and pollen development has been proven in Arabidopsis and rice, it was unknown whether such a mechanism is conserved in the Solanaceae. Here, we examined anther development of two economically important dicots, tobacco (*Nicotiana benthamiana*) and tomato (*Lycopersicon esculentum, cv. ‘moneymaker’*), in detail. We identified the same ROS amplitude during anther development in these two species and found that dynamic ROS level correlates with the initiation and progression of tapetal PCD. We further showed that manipulating ROS levels during anther development severely impaired pollen development, resulting in partial male sterility. Finally, real-time quantitative PCRs (qPCRs) showed that several tobacco and tomato *RBOH*s, encoding NADPH oxidases, are preferentially expressed in anthers. Manipulating the ROS amplitude through genetic interference of *RBOH*s therefore may provide a practical way to generate male sterile plants in the Solanaceae.

## Results

### Anther development of tobacco and tomato accompanies with dynamic ROS levels

To determine whether anthers of tobacco and tomato exhibited dynamic ROS amplitude during development, similar to that shown in Arabidopsis [22], we first classified anthers into six groups (Additional file 1: Table S1), based on the correlation of developmental stages from transverse-sections of anthers with the sizes of floral buds and anthers (Additional file 1: Figures S1 and S2). These six groups represent pre-meiosis, meiosis, tetrad, microspore, mitosis, and dehiscence (Additional file 1: Figures S1, S2 and Table S1).

**Fig. 1:**
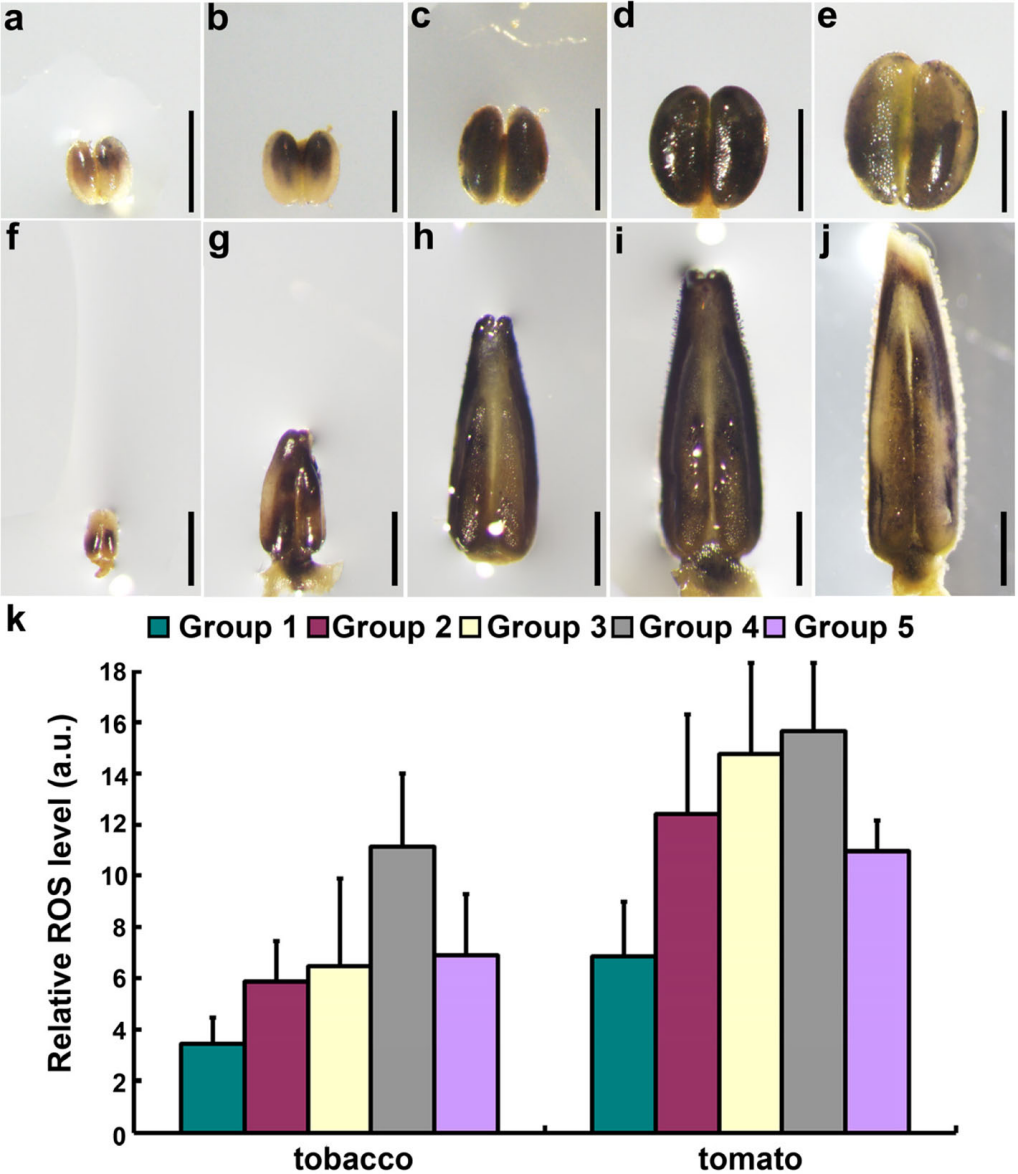
Temporal ROS levels during anther development in tobacco and tomato. **a**–**j** Representative NBT staining of tobacco (**a**–**e**) or tomato (**f**–**j**) anthers during development. In total, 20–25 anthers at each development stage from three independent batches of plants were analyzed and similar results were obtained. **k** Relative ROS levels in tobacco or tomato anthers at each developmental stage based on H2DCF-DA staining of ROS. Results shown are means + standard deviation (SD, *N* = 20). a.u., arbitrary fluorescent unit. Classification of anther groups is based on anther sizing described in Additional file 1: Table S1. *Bars*, 800 μm for (**a**–**e**); 1000 μm for (**f**–**j**)

Next, we treated anthers at different developmental stages with either nitrotetrazolium blue chloride (NBT) or 2′,7′-dichlorodihydrofluorescein diacetate (H_2_DCF-DA) dye, both of which are proxies for ROS levels [21, 22, 24–26]. In tobacco anthers, ROS staining by NBT was detected as early as the pre-meiosis stage (Fig. 1a and k), gradually increased during the meiotic stage (Fig. 1b, c, and k) and was highest at the later microspore stage (Fig. 1d and k). Starting from the mitosis stage to anther dehiscence, ROS signals decreased (Fig. 1d–k). Similar ROS amplitude was observed during tomato anther development (Fig. 1f–k). Taken together, these data showed dynamic ROS amplitude during anther development in both tobacco and tomato, similar to that reported in Arabidopsis [22].

### Pollen development in tobacco anthers

To examine the timing and progression of tapetal PCD during pollen development, it was necessary to finely dissect pollen developmental stages. Unlike Arabidopsis and rice, for which extensive studies have been conducted regarding pollen developmental processes within anthers [27, 28], pollen development of both tobacco and tomato has not been thoroughly described, although there are a few histological studies [29, 30].

**Fig. 2:**
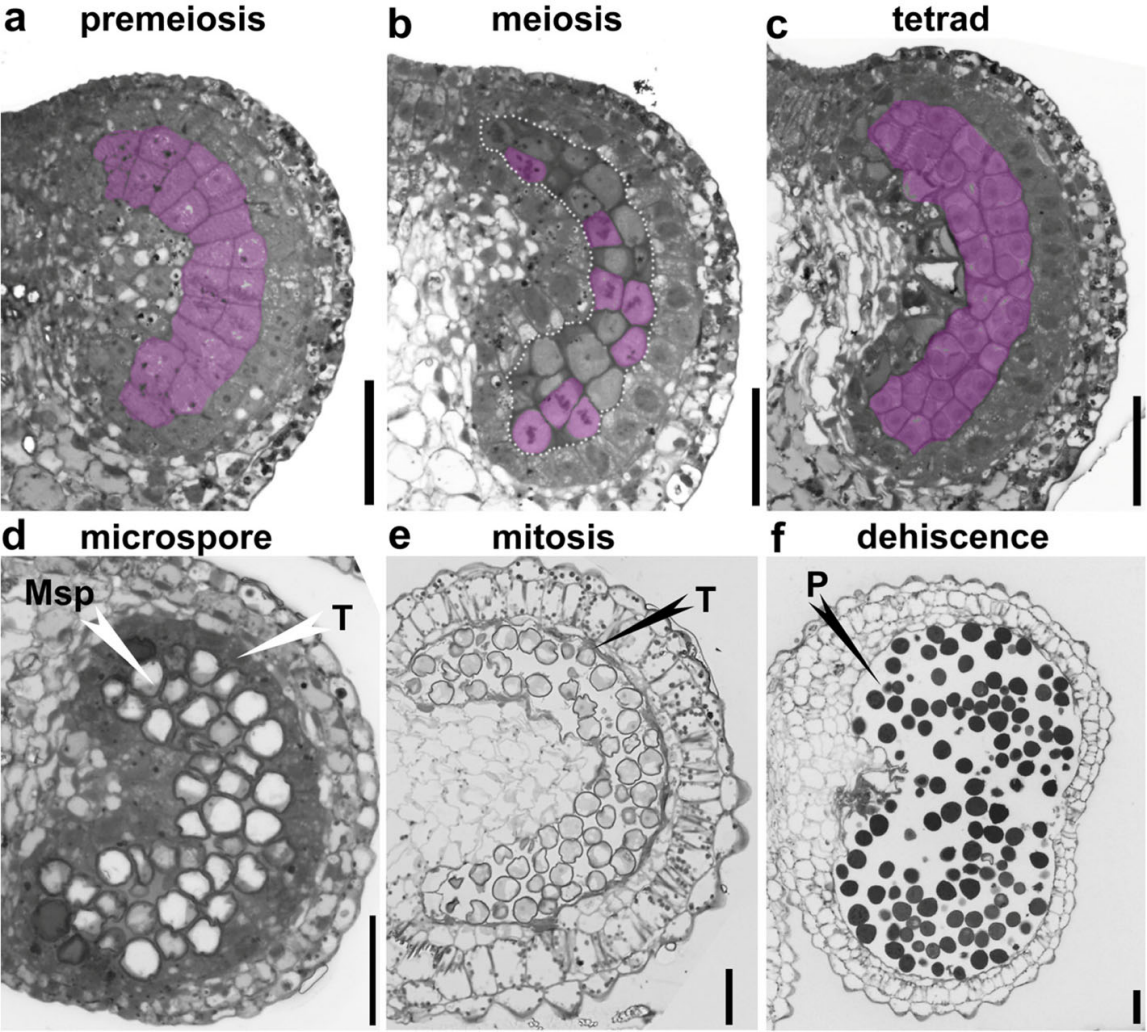
Histological characterization of anther development in tobacco (*Nicotiana Benthamiana*). Semi-thin transverse sections of tobacco anthers at pre-meiosis (**a**), meiosis (**b**), tetrad (**c**), microspore (**d**), mitosis (**e**), and dehiscence (**f**) stages. *Pink*-shaded cells in (**a**–**c**) are sporogeneous cells (**a**), pollen mother cells (PMC) at metaphase (**b**), or tetrads (**c**). Areas highlighted by the dotted line in (**b**) are a cluster of PMCs. Msp, microspores; P, pollen; T, tapetum. *Bars*, 50 μm

Therefore, we first performed semi-thin transverse sections of tobacco (*Nicotiana benthamiana*) anthers of different sizes based on a roughly-defined developmental staging (Additional file 1: Figure S1 and Table S1). At the pre-meiosis stage, pollen sacs were established with four anther cell layers (Fig. 2a). The layers, from the outermost to the innermost, are epidermis, endothecium, middle layer, and tapetum, which surround microspore mother cells (MMC) (Fig. 2a). Nuclei of tapetal cells gradually enlarged and cytoplasm condensed when the MMCs entered the meiotic stage (Fig. 2b). From this stage on, tobacco anthers enlarged rapidly (Additional file 1: Figure S1). After completion of meiosis, tetrads of microspores (MSPs) enclosed by a thick callose wall were formed (Fig. 2c). During the tetrad stage, tapetal cells underwent nuclear division to form bi-nucleate or poly-nucleate cells, with an increase in cell volume (Fig. 2c). MSPs were finally released from tetrads at the microspore stage and became vacuolated (Fig. 2d). At the same time, the tapetal cells condensed and shrank (Fig. 2d), indicating release of their content to pollen sacs. At the mitotic stage, the tapetum was reduced to a very thin layer (Fig. 2e). At this stage the endothecium expanded (Fig. 2e). Finally, at the dehiscence stage, the septum and stomium were disrupted to release mature pollen grains and the tapetal layer was no longer visible (Fig. 2f).

**Fig. 3:**
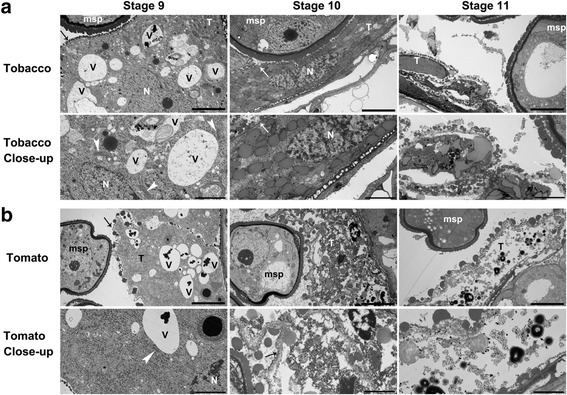
Tomato and tobacco anthers at stage 9–11 showing the degeneration process of the tapetum. Transmission electron micrographs (TEMs) of tobacco (**a**) or tomato (**b**) anthers at anther developmental stage 9–11. *Arrowheads* point at intact Golgi apparatus. Arrows point at the plasma membrane of the tapetal cells. *Asterisks* highlight mitochondria. Msp, microspores; N, nucleus; T, tapetum; V, vacuoles. *Bars* = 5 μm for overall images, i.e. the *top panel* images in (**a**–**b**); = 2 μm for close-up images, i.e. the bottom panel images in (**a**–**b**)

To verify the results from the semi-thin sections and to better evaluate the cellular changes in tapetal cells, we performed transmission electron microscopy (TEM) of tobacco anthers at different developmental stages. Consistent with the results obtained by semi-thin sections, tapetal cells maintained cellular integrity at stage 9, in that mitochondria and the Golgi apparatus were visible (Fig. 3a). At the later microspore stage, i.e. stage 10, the plasma membrane (PM) of tapetal cells was intact although the nuclei started to show signs of disintegration (Fig. 3a). Only at stage 11, was the cellular integrity of the tapetal cells disrupted and cell contents were released into the pollen sac (Fig. 3a), indicating tapetal degeneration.

### Tapetal PCD in tobacco

**Fig. 4:**
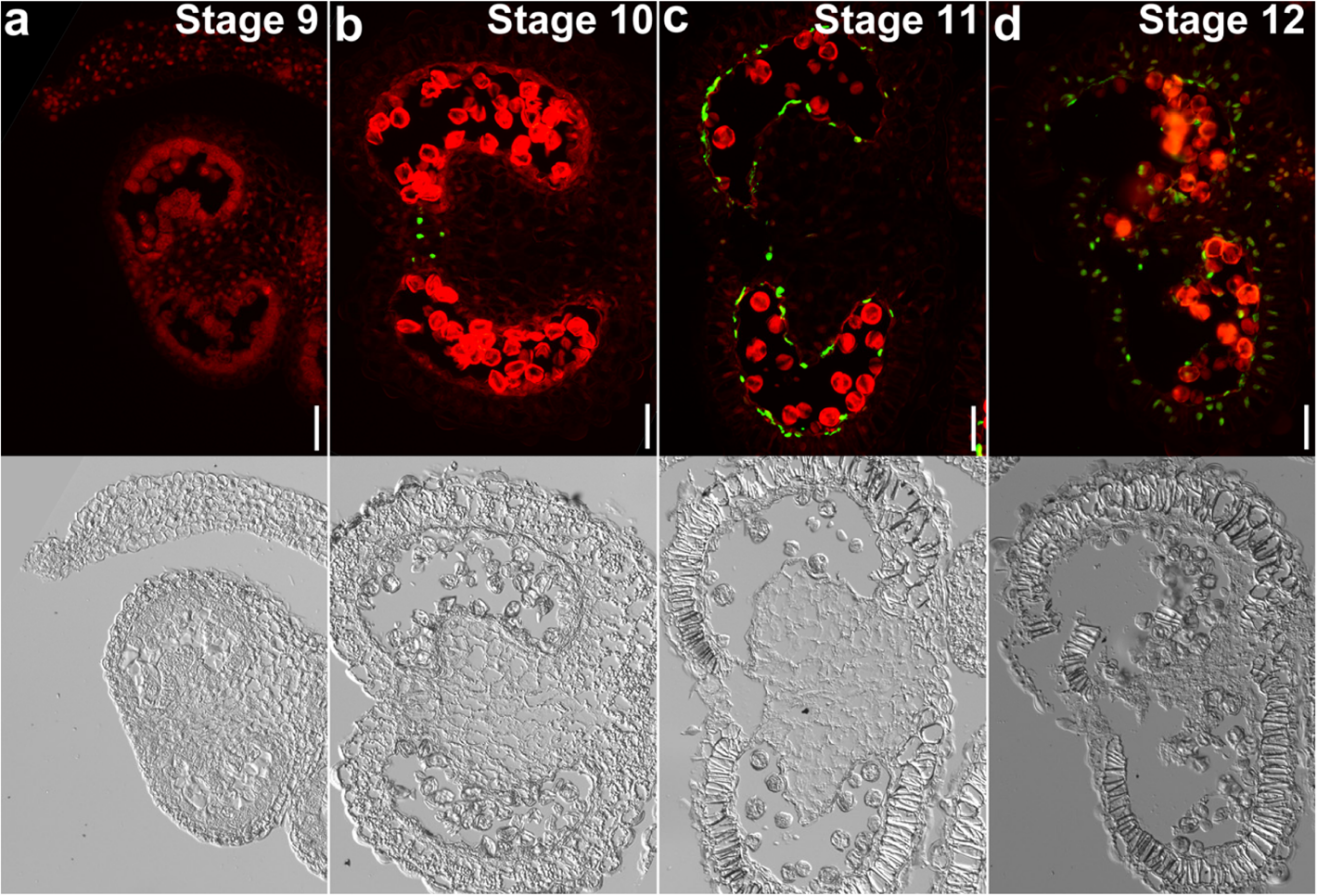
Tapetal PCD during tobacco anther development by TUNEL assays. **a**–**d** Fluorescence microscope of cross-sections of tobacco anthers at stage 9 (**a**), stage 10 (**b**), stage 11 (**c**), and stage 12 (**d**). *Green* fluorescence indicates TUNEL-positive signals while *red* fluorescence indicates propidium iodide (PI) staining. Corresponding transmission images are placed below the fluorescence images. *Bars*, 50 μm

Having established the detailed histological events leading to pollen development, we performed terminal deoxynucleotidyl transferase–mediated dUTP nick-end labeling (TUNEL) assays on tobacco anthers at different developmental stages to determine the timing and progression of tapetal PCD. Positive TUNEL signals indicate the occurrence of PCD [22]. No TUNEL signals were detected at stage 9 in tobacco anthers (Fig. 4a), which corresponds to the late tetrad or early microspore stage. At stage 10, which corresponds to the late microspore stage, positive TUNEL signals were detected in septum cells (Fig. 4b), suggesting that the degeneration of the septum occurred earlier than in Arabidopsis [22]. However, no TUNEL signals were detected in the tapetal layer at this stage (Fig. 4b), suggesting that tapetal PCD of tobacco anthers occurred much later than in Arabidopsis [22]. Massive TUNEL signals were detected at stage 11 (Fig. 4c), which corresponds to the mitotic stage. At this stage, septum cells were completely degenerated (Fig. 4c). At stage 12, when anthers began to dehisce, the other cell layers in anthers, such as endothecium, also had TUNEL signals (Fig. 4d), suggesting extensive cell death of in the sporophytic anther tissues. These results showed that tapetal PCD of tobacco anthers initiated at the late microspore or early mitotic stage and progressed rapidly.

### Pollen development in tomato

**Fig. 5:**
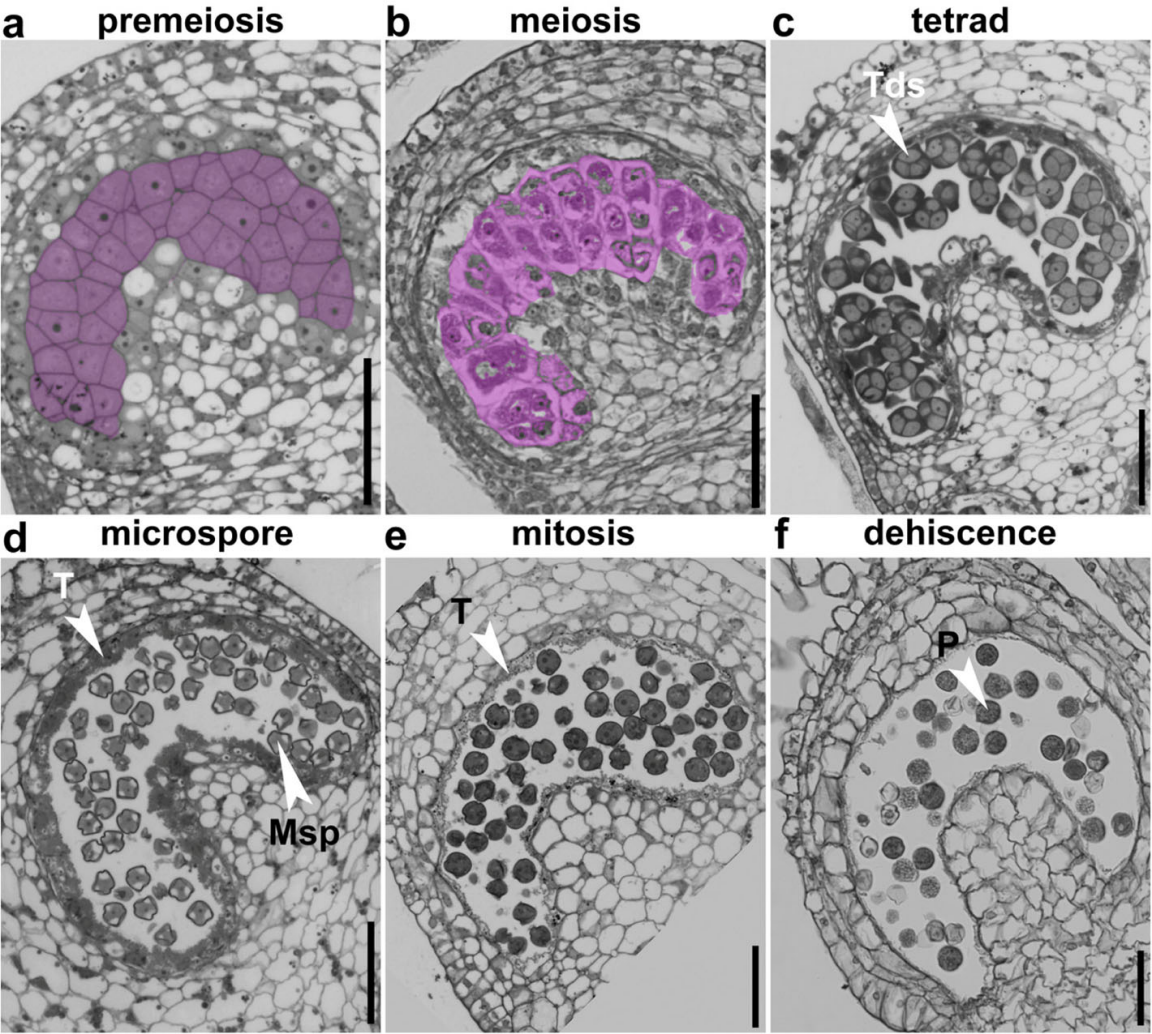
Histological characterization of anther development in tomato (*Solanum lycopersicum*). Semi-thin transverse sections of tobacco anthers at pre-meiosis (**a**), meiosis (**b**), tetrad (**c**), microspore (**d**), mitosis (**e**), and dehiscence (**f**) stages. Pink-shaded cells in (**a**–**b**) are sporogeneous cells (**a**) or pollen mother cells (PMC) at metaphase (**b**). Msp, microspores; P, pollen; Tds, tetrads; T, tapetum. *Bars*, 50 μm

We also applied semi-thin transverse sections on anthers of tomato (*Lycopersicon esculentum*) to determine the histological processes during its pollen development. At the pre-meiosis stage, archesporial tissues were established with MMCs visible (Fig. 5a). Afterwards, MMCs started meiosis with callose deposited around them (Fig. 5b). At this stage, tapetal cells were bi-nucleate or poly-nucleate (Fig. 5b). The end of meiosis led to the formation of tetrads within pollen sacs, surrounded by thick callose wall (Fig. 5c). Unlike in tobacco, at this stage the tapetal layer of tomato became thinner and more condensed (Fig. 5c). During the microspore stage, MSPs were released and became vacuolated (Fig. 5d). The plasma membrane (PM) of the tapetal cells became disintegrated (Fig. 5d), suggesting progression of cell death. At the mitotic stage, the pollen cytoplasm was condensed rather than vacuolated (Fig. 5e), and the tapetal layer became thin and fragmented (Fig. 5e). Finally, tomato anthers dehisced to release mature pollen, although a very thin layer of tapetum, appressed to the middle layer, was still visible (Fig. 5f). TEM studies verified that the degeneration of tapetal cells in tomato started earlier, during the microspore stage (Fig. 3b), as at stage 9, the tapetal cells already showed signs of cellular disintegration, i.e. the PM of tapetal cells, albeit intact, was convoluted (Fig. 3b), and the nuclei also showed signs of degeneration (Fig. 3b). At the late microspore stage, i.e. stage 10, the tapetal cells of tomato anthers had already degenerated, containing hardly any intracellular organelles (Fig. 3b). Starting at the microspore stage, and verified by TEM, which showed disintegrated PM (Fig. 3). Plenty of Ubisch bodies associated with the cell surface, which was not even continuous (Fig. 3b). At the stage 11, tapetal cells had degenerated (Fig. 3b).

### Tapetal PCD in tomato

**Fig. 6:**
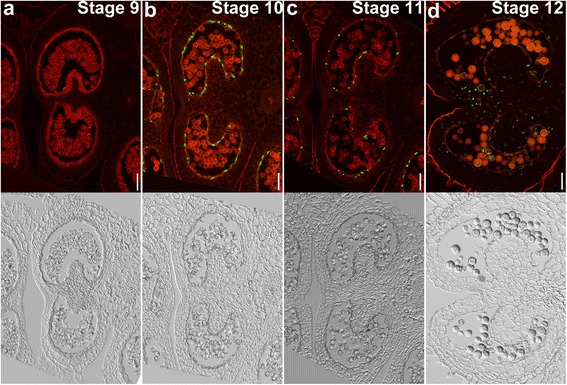
Tapetal PCD during tomato anther development by TUNEL assays. **a**–**d** Fluorescence microscope of cross-sections of tomato anthers at stage 9 (**a**), stage 10 (**b**), stage 11 (**c**), and stage 12 (**d**). *Green* fluorescence indicates TUNEL-positive signals while *red* fluorescence indicates PI staining. Corresponding transmission images are placed below the fluorescence images. *Bars*, 50 μm

The differential histology between tomato and tobacco during pollen development suggested distinct tapetal PCD. To test this hypothesis, we performed TUNEL assays on tomato anthers at different developmental stages. Indeed, tapetal PCD in tomato resembled that in Arabidopsis, and not that of tobacco, in that it initiated at stage 10, i.e. at late microspore stage (Fig. 6b). TUNEL signals were detected from stage 10 to at late as stage 12 (Fig. 6b–d). Few TUNEL signals at the tapetal layer were visible at stage 12 (Fig. 6d), indicating the completion of tapetal cell degeneration. At stage 12, septum cells and the endothecium had degenerated and exhibited TUNEL signals (Fig. 6d), indicating the start of anther dehiscence. These results showed that tapetal PCD of tomato initiated earlier but progressed more slowly than in tobacco.

### Pharmacological interference of ROS amplitude impaired pollen development

**Fig. 7:**
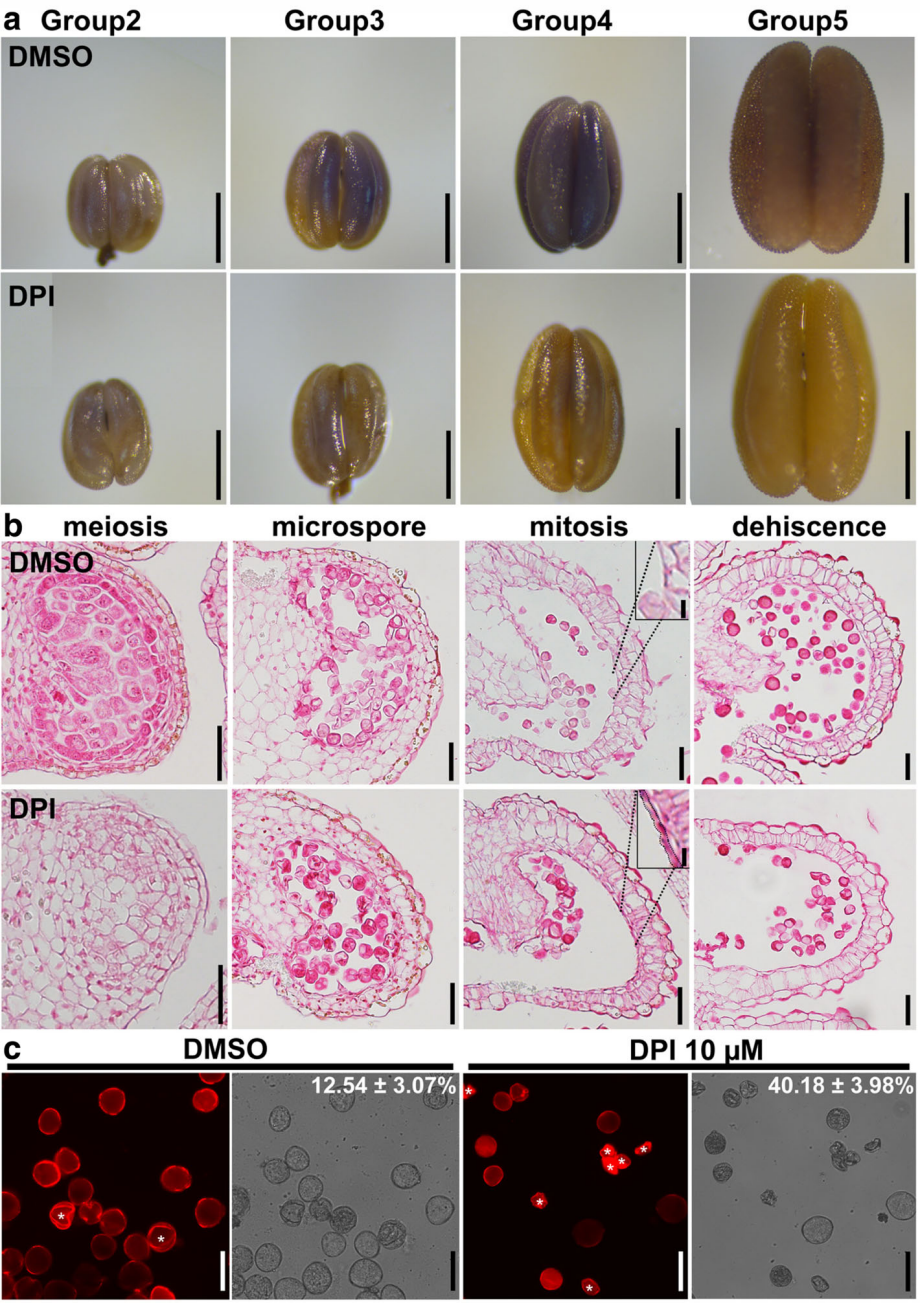
Pharmacological interference of ROS amplitude compromised tapetal degeneration and pollen development. **a** Representative NBT staining of tobacco anthers at different developmental stages from floral buds treated with DMSO or with 10 μM DPI. **b** Transverse sections of tobacco anthers at the stage of meiosis, microspore, mitosis, or dehiscence from DMSO- or DPI-treated flowering buds. *Arrowheads* point to the tapetal layer. Insets are close-ups showing the tapetal layer (highlighted with *doted lines*, DPI-treated) or its absence (DMSO-treated). **c** PI staining of mature pollen grains released from tobacco flowers treated with DMSO or with DPI during anther development. Transmission image is shown at the right side of its corresponding fluorescence image. *Asterisks* indicate aborted pollen grains. Numbers at the top are means ± standard deviation (SD, *N* = 20–25). DMSO and DPI treatments are significantly different (*t*-test, *P* < 0.01). *Bars*, 500 μm for (**a**); 50 μm for (**b**, **c**); 10 μm for (**b** inset)

The dynamic ROS amplitude in tobacco and tomato anthers (Fig. 1) during development resembled that in Arabidopsis [22]. Because Arabidopsis ROS is critical for the timing and progression of tapetal PCD [22], we wondered whether it was also true for these species. To test that, we applied 10 μm diphenyleneiodonium chloride (DPI) on tobacco anthers to scavenge ROS [31]. Anthers subjected to DPI or DMSO (mock) treatment were collected at different timepoints representing various developmental stages, and then stained with NBT to determine the ROS level. Indeed, the ROS level decreased from the tetrad to mitosis stages upon DPI treatment, relative to the levels in anther treated with DMSO (Fig. 7a). To determine the extent of tapetal degeneration after DPI treatment, we next performed histological analysis by using transverse sections of anthers. The tapetal layer of tobacco anthers treated with DMSO had almost disappeared at stage 11 (Fig. 7b), but anthers treated with DPI still contained a thin layer of tapetal cells (Fig. 7b). Delayed tapetal degeneration after DPI treatment correlates well with a reduced ROS amplitude. Finally, to determine the effect of reduced ROS amplitude on pollen development, we stained mature pollen grains released from DPI- and DMSO-treated anthers. Consistently, significantly more aborted pollen grains were present in DPI-treated anthers (Fig. 7c). The results suggest that reduced ROS amplitude delays tapetal degeneration and impairs pollen development.

### Anther-preferential expression of *RBOH*s in tobacco and tomato

ROS can be generated by mitochondria, peroxisomes, the cytoplasm, and the plasma membrane (PM) [32]. Extensive studies have demonstrated the critical roles of the PM-localized NADPH oxidases in ROS signaling [20, 33], including tapetal PCD [22]. We were thus interested in determining whether expression changes of the NADPH oxidase-encoding genes, *RBOH*s, contribute to the dynamic ROS amplitude during anther development in tobacco and tomato.

**Fig. 8:**
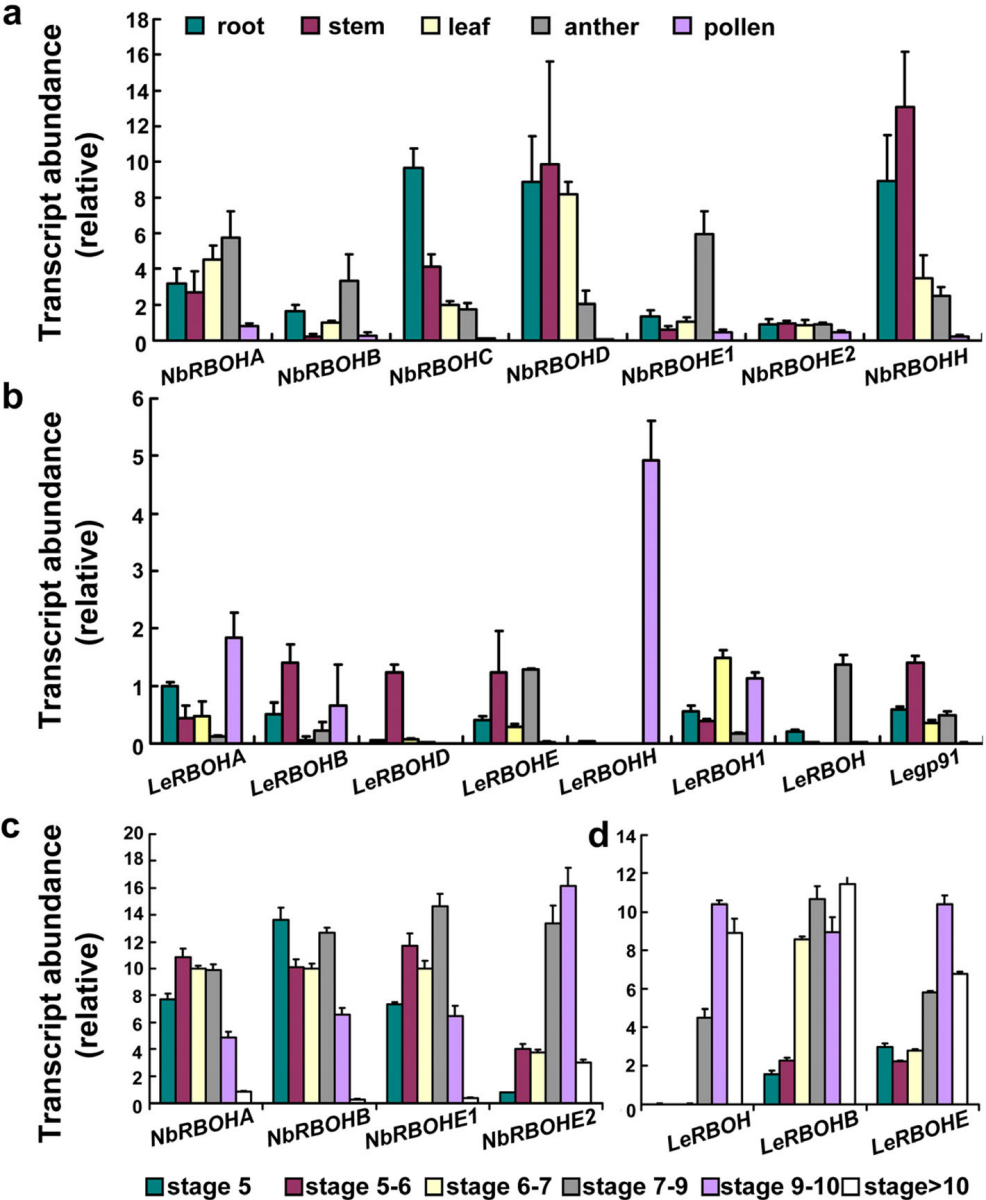
Transcript abundance of tobacco or tomatao *RBOH*s in various tissues or anther development stages by real-time quantitative PCRs. **a**–**b** Transcript abundance of tobacco (**a**) or tomato (**b**) *RBOH*s in various tissues. **c**–**d** Transcript abundance of tobacco (**c**) or tomato (**d**) *RBOH*s in anthers at various developmental stages. RNAs used in (**a**–**b**) were extracted from root, stem, leaf, mature anther, or pollen. RNAs used in (**c**–**d**) were extracted from anthers at different developmental stages. Classification of anther groups is based on anther sizing described in Additional file 1: Table S1. Data shown are means + SE (*N* = 3)

We first performed BLAST searches at NCBI to identify tobacco and tomato *RBOH*s using the Arabidopsis *RBOH* sequences as queries. We identified seven tobacco and eight tomato *RBOH*s (Additional file 1: Figure S4). Of these, only *NbRBOHA* and *NbRBOHB*, have been studied, and in regard to elicitor-induced stomatal closure and the hypersensitive response [34, 35]. Quantitative real-time PCRs (qPCRs) were performed to examine the expression of these genes in various tobacco and tomato tissues, including roots, stem, leaves, anthers, and pollen (Fig. 8a, b). Among the 7 tobacco *RBOH*s, *NbRBOHA, NbRBOHB, NbRBOHE1*, and *NbRBOHE2* were preferentially expressed in anthers (Fig. 8a), and *LeRBOHB*, *LeRBOHE*, and *LeRBOH* were preferentially expressed in anthers (Fig. 8b). We then extracted mRNAs from anthers at different developmental stages (Additional file 1: Table S1) and examined the temporal expression of these anther-preferential genes by qPCR. Indeed, *NbRBOHE1*, *NbRBOHE2*, *LeRBOHE*, and *LeRBOH* showed a temporal expression pattern in anther development (Fig. 8c, d), similar to that of Arabidopsis *RBOHE* [22], whose function is critical for the dynamic ROS amplitude in Arabidopsis [22]. The spatiotemporal expression of these four genes in tobacco and tomato implies their involvement in contributing to the dynamic ROS amplitude during anther development.

## Discussion

Another developmental stages of tobacco and tomato were divided into 20 stages beginning from stamen primordial formation to mature pollen [29, 30]. Based on the clearly defined anther developmental stages in Arabidopsis [27] and histological analyses (Figs. 2 and 5), we classified tobacco and tomato anther development into six major stages, i.e. pre-meiosis, meiosis, tetrad, microspore, mitosis, and dehiscence. These stages are characterized by major events occurring during pollen maturation. Anther development of tomato and tobacco differs slightly from that in Arabidopsis or rice, especially regarding tapetal cells. In the crescent-shaped anther locules of tobacco and tomato anthers, the inner, i.e. proximal tapetal cells, were more vacuolated than the outer, i.e. distal tapetal cells, which are more electron-dense (Figs. 2 and 5). By comparison, in the round anther locules of Arabidopsis and rice, tapetal cells are not dimorphic [27, 28].

By using NBT and H_2_DCF-DA staining of ROS in anthers of tobacco and tomato at various developmental stages, we demonstrated that dynamic ROS amplitude during anther development. Although the gradual increase of ROS during anther development occurs slightly earlier in tomato (early microspore stage) than in tobacco (Fig. 1, Additional file 1: Figure S3), the highest level of ROS was detected at the microspore stage in both species (Fig. 1), suggesting that conserved ROS amplitude during anther development. Although dynamic ROS levels were reported in developing anthers of now four different plant species, slight differences exist regarding timing and amplitude. In both Arabidopsis and rice, the ROS level rapidly increased at stage 8 and reached the highest level at stage 9 i [21, 22]. After that, it decreased rapidly, achieving a basal level at stage 11 [21, 22]. By comparison, in tomato and tobacco the ROS level slowly and gradually increased starting from stage 5–6 (Fig. 1, Additional file 1: Figure S3), reaching the highest at stage 8–9, followed by a decrease starting from stage 10 (Fig. 1).

By using transverse sections as well as TUNEL assays, we analyzed the initiation and progression of tapetal PCD in tobacco and tomato anthers. As reported for Arabidopsis [22], the tapetal layer of tomato anthers began condensation as early as the tetrad stage (Fig. 5). Soon after the release of microspores (Fig. 5), positive TUNEL signals were detected in the tapetal layers of tomato anthers (Fig. 6), suggesting massive tapetal PCD. At the mitotic stage, the PM of tapetal cells lost its integrity (Fig. 5), indicating the end of tapetal degeneration. By contrast, tapetal cells of tobacco anthers contained enlarged nuclei and cell volume at the tetrad stage (Fig. 2). Cell condensation was observed only at late microspore stage (Fig. 2), which led to the appearance of TUNEL signals (Fig. 4). Massive tapetal cell death was detected at the mitotic stage in tobacco anthers (Fig. 4), consistent with a rapid reduction of the tapetal layer at the same stage based on histological analysis (Fig. 2). In comparison, tapetal PCD started at the dyad stage (stage 8a) and finished at the middle microspore stage (stage 9) in rice anthers [36], indicating that distinct timing or progression of tapetal PCD among different plant species is possible.

The slight difference in the timing and progression of tapetal PCD between tobacco and tomato correlates well with the dynamic ROS amplitude. The increase of ROS in tomato anthers started earlier but progressed slowly, consistent with the early initiation and long duration of tapetal PCD (Fig. 6). In comparison, ROS in tobacco anthers increased abruptly at the mitotic stage, followed by a sudden drop (Fig. 1, Additional file 1: Figure S3), consistent with the late initiation and short duration of tapetal PCD (Fig. 4). Pharmacologically scavenging ROS by applying DPI during anther development severely compromised the ROS amplitude (Fig. 7). Consequently, tapetal degeneration was delayed and pollen development impaired (Fig. 7). These results suggest that the dynamic ROS amplitude during anther development is intimately linked with the initiation and progression of tapetal PCD.

Although ROS can be produced from different intracellular sources [32], we consider the PM-localized NADPH oxidases essential for the dynamic ROS amplitude during anther development. First, Arabidopsis *RBOH*s have been demonstrated to mediate the dynamic ROS amplitude, and mutations in these genes interfered with tapetal PCD [22]. Second, as PM-associated ROS source [37, 38], NADPH oxidases are localized at the interface between developing microspores and the tapetal cells, and therefore would be good candidates to facilitate intercellular communication. Third, among several *RBOH*s in tobacco and tomato, we identified a few whose preferential expression in anthers suggests their potential functionality during tapetal PCD (Fig. 8). Indeed, the expression of these *RBOH*s not only showed tissue specificity but also displayed a temporal expression pattern during anther development (Fig. 8), correlating with the temporal pattern of ROS (Fig. 8). Reverse genetics by the newly developed genome-editing technologies, such as CRISPR-Cas9 [39], on these anther-preferential *RBOH*s may provide a feasible way to generate male-sterile tobacco and tomato plants.

## Methods

### Plant materials and growth conditions

Tobacco (*Nicotiana benthamiana*) and tomato (*Lycopersicon esculentum*, *cv.* ‘moneymaker’) were grown in an incubator at 25/20 °C (day/night) under long days.

### RNA-extraction and real-time quantitative PCR (qPCRs)

Total RNAs were extracted from various tissues or anthers at different developmental stages of tobacco and tomato using the Ultrapure RNA kit according to the manufacturer’s instructions (CWBIO). Reverse transcription was performed using Reverse Transcriptase M-MLV (Takara). The qRT-PCR analyses were performed with the BioRad CFX96 real-time system using SYBR Green real-time PCR master mix (Toyobo) as described [40]. Sizing of anthers from tobacco or tomato was used to determine developmental stages, as shown in Additional file 1: Table S1. All primers used are listed in Additional file 1: Table S2.

### TUNEL assays

TUNEL assays (in situ nick-end labeling of nuclear DNA fragmentation) were performed using the In Situ Cell Death Detection Kit TUNEL system (Roche) according to the supplier’s instructions. Samples were analyzed with Axio Observer D1 microscope equipped with a CCD camera (Zeiss). Emission/excitation for the TUNEL signals and PI signals are 488 nm/505–550 nm and 561 nm/575–650 nm, respectively.

### Histology of anthers and histochemical assays for ROS

Semi-thin transverse sections as well as TEMs of floral buds at different development stages were performed as described [41]. NBT staining [21] and H_2_DCFDA staining of anthers [42] were performed as described. Fluorescence imaging of H_2_DCFDA stained anthers was performed with an Axio Observer D1 microscope equipped with a CCD camera (Zeiss) at the same parameters for all samples to facilitate comparative quantification. Anthers were classified into five groups based on their sizes as described in Additional file 1: Table S1. Three independent experiments were conducted, and each experiment involved 20 to 25 anthers in one group. Fluorescence intensity of whole anthers was quantified using ImageJ software (http://rsbweb.nih.gov/ij/).

### DPI treatment

DPI was dissolved in DMSO to make a 10 mM stock solution. The stock solution was diluted with 0.02% (*w*/*v*) Triton X-100 as a 10 μM working solution. Same dilution was made for DMSO as the control. One sepal of floral buds corresponding to anther developmental stage 5–7, as day 0 (D0), was removed. DPI or DMSO was sprayed on the floral buds once per day for 5 days until anther stage 10–11. Anthers were harvested at D0, D2, D4, D6, and D8, which corresponds to the stage of meiosis, tetrad, microspore, mitosis, and dehiscence, respectively, for NBT staining and transverse sections. Pollen grains from anthers harvested at D8 were stained with PI to determine pollen viability.

### Phylogenetic analysis of RBOHs in Arabidopsis, tobacco, and tomato

Protein sequences of Arabidopsis RBOHs were obtained from the TAIR website (http://www.arabidopsis.org/). Protein sequences of Arabidopsis RBOHs were used in BLAST search at NCBI to identify tobacco RBOHs. Protein sequences of tomato RBOHs were obtained from the tomato genomic website (http://www.solgenomics.net/). The software Vector NTI (Invitrogen) was used to perform phylogenetic analysis.

## Conclusions

In this study, we demonstrated that pollen development of two economically important dicot species, tobacco and tomato, requires dynamic ROS amplitude during anther development. Such ROS amplitude correlates with the onset and progression of tapetal PCD. Furthermore, we provide evidence supporting a key role of anther-preferentially expressed *RBOH* genes in the generation of tapetal ROS amplitude. Results presented here provide theoretical bases to generate commercially valuable male sterile plants.

## Additional file

Additional file 1: The following materials are available in the online version of this article. **Figure S1.** Staging of tobacco anther development. **Figure S2.** Staging of tomato anther development. **Figure S3.** Temporal ROS levels during anther development in tobacco and tomato by H_2_DCF-DA staining. **Figure S4.** Phylogenetic analysis between Arabidopsis, tobacco and tomato RBOHs. **Table S1.** Sizing and major events during tobacco or tomato anther development. **Table S2.** Oligos used in real-time PCRs. (PDF 2041 kb)
