# Supplementary material for: Reactive oxygen species mediate tapetal programmed cell death in tobacco and tomato
Source: BMC Plant Biol. 2017 Apr 20;17:76. doi: 10.1186/s12870-017-1025-3 (PMC5399379; doi:10.1186/s12870-017-1025-3)
Supplement: Additional file 1: — The following materials are available in the online version of this article. Figure S1. Staging of tobacco anther development. Figure S2. Staging of tomato anther development. Figure S3. Temporal ROS levels during anther development in tobacco and tomato by H2DCF-DA staining. Figure S4. Phylogenetic analysis between Arabidopsis, tobacco and tomato RBOHs. Table S1. Sizing and major events during tobacco or tomato anther development. Table S2. Oligos used in real-time PCRs. (PDF 2041 kb) [file 12870_2017_1025_MOESM1_ESM.pdf]

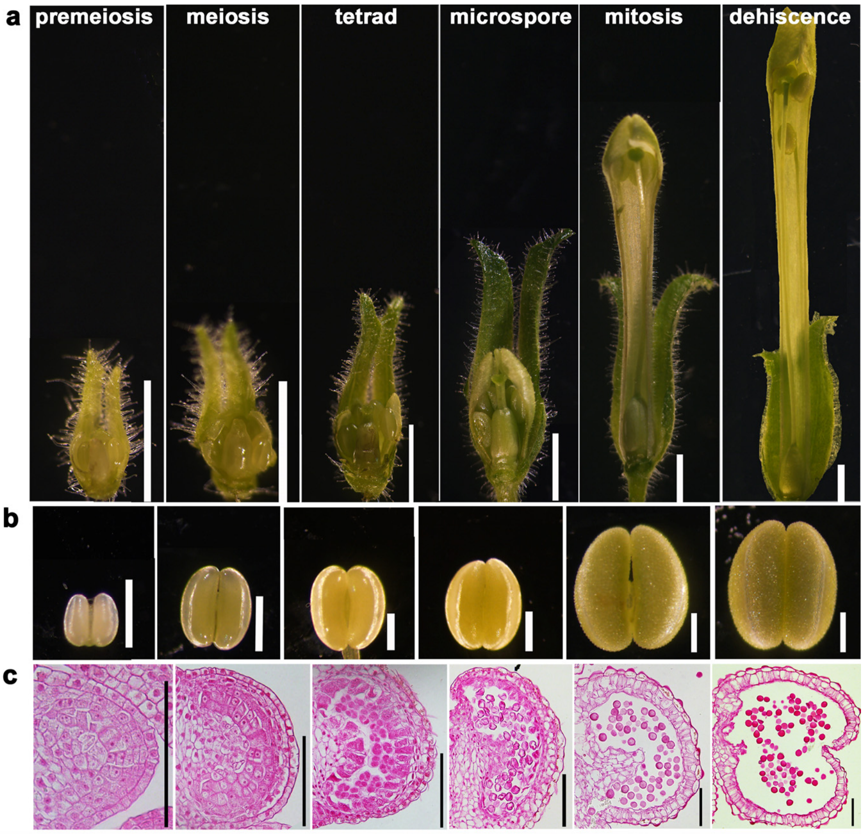

Supplemental Figure 1. Staging of tobacco anther development.

(a) Representative floral buds with exposed inner whorls at various developmental stages. (b) Representative anthers at various developmental stages. (c) Transverse sections of anthers at the stage of premeiosis, meiosis, tetrad, microspore, mitosis, or dehiscence. Bars, 2 mm for (a), 500 µm for (b), and 100 µm for (c).

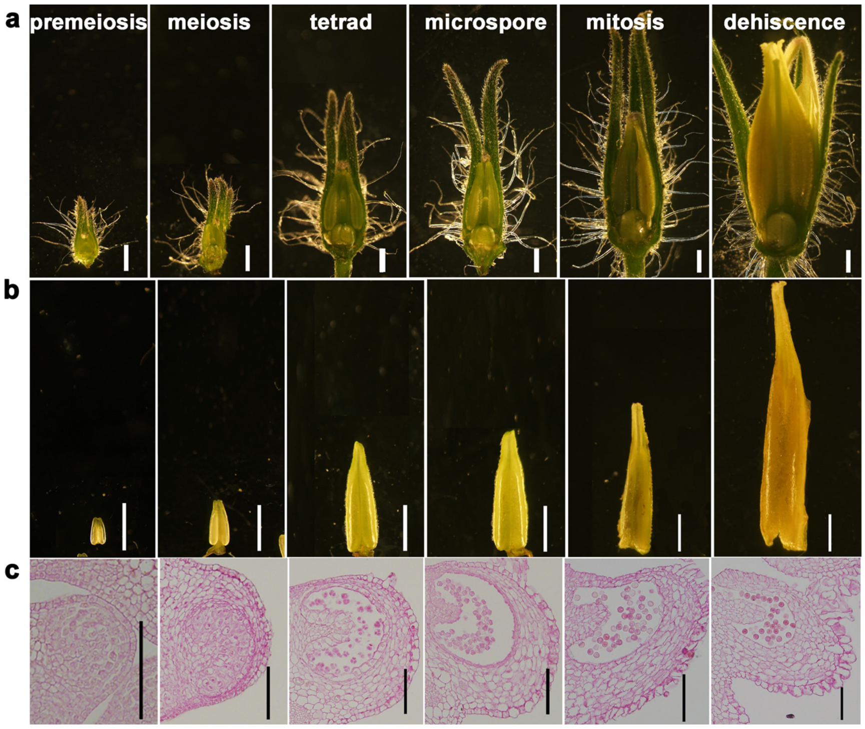

Supplemental Figure 2. Staging of tomato anther development.

(a) Representative floral buds with exposed inner whorls at various developmental stages. (b) Representative anthers at various developmental stages. (c) Transverse sections of anthers at the stage of premeiosis, meiosis, tetrad, microspore, mitosis, or dehiscence. Bars, 1 mm for (a, b), and 100 μm for (c).

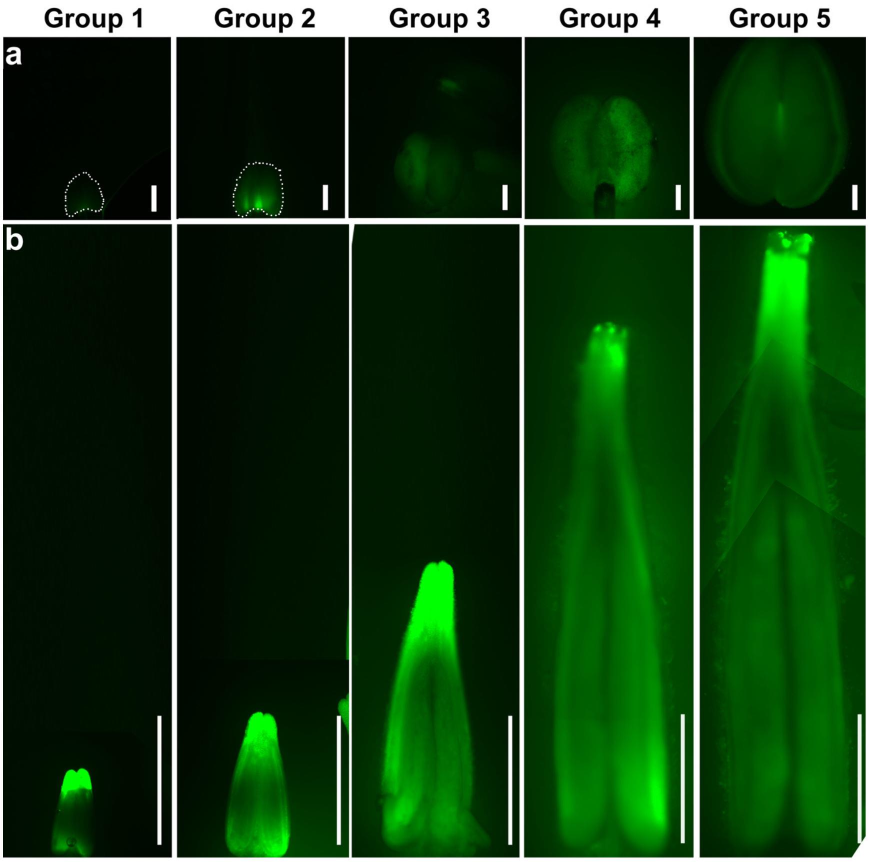

Supplemental Figure 3. Temporal ROS levels during anther development in tobacco and tomato by H_2DCF-DA staining.

(a-b) Representative H_2DCF-DA staining of tobacco (a) or tomato (b) anthers during development. In total 20-25 anthers at each development stage from three independent batches of plants were analyzed and similar results were obtained. Bars, 200 µm for (a), 1000 µm for (b).

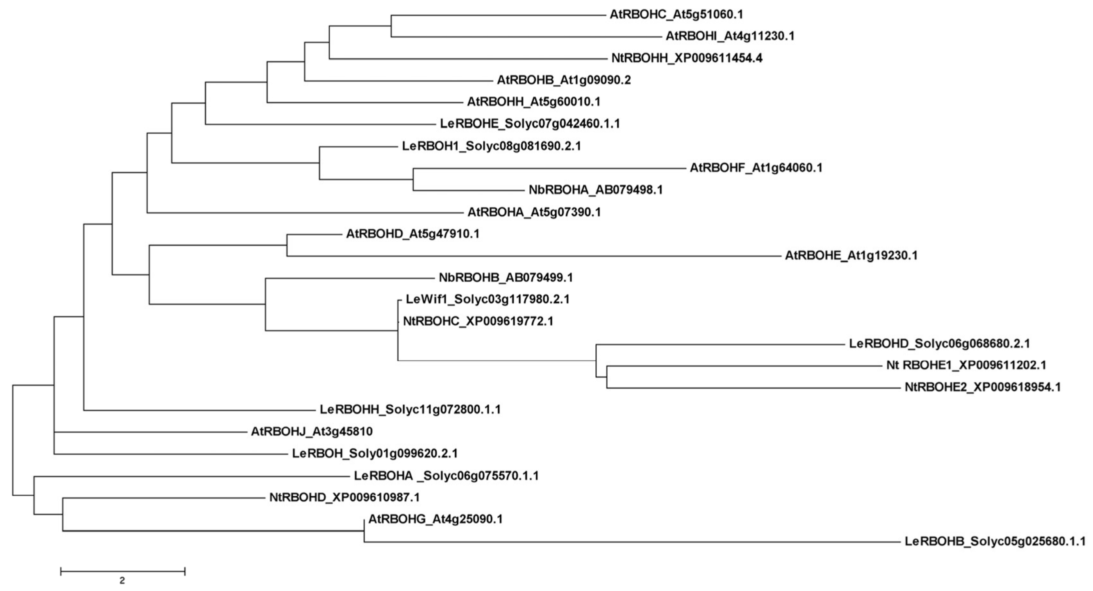

Supplemental Figure 4. Phylogenetic analysis between Arabidopsis, tobacco and tomato RBOHs. The tobacco, tomato and Arabidopsis protein sequence analysis used MEGA software. Arabidopsis protein sequence number obtained from tair website, tobacco protein sequence obtained from NCBI data, tomato protein sequence obtained from tomato website. Sequence data from this article can be found in the GenBank databases under the following accession numbers: At5g07390 for *AtRBOHA*; At1g09090 for *AtRBOHB*; At5g51060 for *AtRBOHC*; At5g47910 for *AtRBOHD*; At1g19230 for *AtRBOHE*; At1g64060 for *AtRBOHF*; At4g25090 for *AtRBOHG*; At5g60010 for *AtRBOHH*; At4g11230 for *AtRBOHI*; At3g45810 for *AtRBOHJ*; AB079498.1 for *NbRBOHA*; AB079499.1 for *NbRBOHB*; XP009619772.1 for *NtRBOHC*; XP009610987.1 for *NtRBOHD*; XP009611202.1 for *NtRBOHE1*; XP009618954.1 for *NtRBOHE2*; XP009611454.4 for *NtRBOHH*; Solyc06g075570.1.1 for *LeRBOHA*; Solyc05g025680.1.1 for *LeRBOHB*; Solyc06g068680.2.1 for *LeRBOHD*; Solyc07g042460.1.1 for *LeRBOHE*; Solyc11g072800.1.1 for *LeRBOHH*; Solyc08g081690.2.1 for *LeRBOH1*; Solyc01g099620.1 for *LeRBOH*; and Solyc03g117980.2.1 for *LeWif1*.

Supplementary Table 1. Sizing and major events during tobacco or tomato anther development.

Stage	Bud length (mm) [a,1]	Anther length (mm) [a,1]	Bud length (mm) [b,1]	Anther length (mm) [b,1]	Event [c]
5	2-2.5	0.25-0.4	2.4-3.5	0.55-1	Premeiosis
6	2.5-5.5	0.4-0.7	3.5-5.5	1.0-2.0	Meiosis
7	5.5-6.5	0.7-0.85	5.5-7	2.0-3.0	Tetrad
8-9	6.5-10	0.85-1.35	7.0-10	3.0-4.5	Microspore
11	10.0-15	1.35-1.5	10.0-11	4.5-5.5	Mitosis
12-13	>15	>1.5	>11	>5.5	Dehiscence

[1] Mean of 20 anthers from five plants.

[a] Tobacco

[b] Tomato

[c] Based on semi-thin transverse anther sections.

Supplementary Table 2. Oligos used in real-time PCRs.

Gene symbol	No. Oligos	Primer sequences (5'-3')
LeACTIN	ZP4402	GGAACTTGAGAAGGAGCCTAAG
	ZP4403	CAACACCAACAGCAACAGTCT
LeRBOHA	ZP4129	CTCCTTCACATTGGCTACAC
	ZP4130	GAGGATGTAGACGAGGACCAG
LeRBOHB	ZP4131	CAGTGCCCAACAATATCCTC
	ZP4132	TCCTGGTGCTGATGTTATAG
LeRBOHD	ZP2948	AAACCAAACAGATAACAACC
	ZP2949	AACAATACAAAACCCAACTA
LeRBOHE	ZP2938	GAGGAATGAATGTACCACTACAC
	ZP2939	GAGATCCCAAAGGACAAATA
LeRBOHH	ZP2944	AAGGTTCATTTCTTCCCATTC
	ZP2945	GGTCATGGTATCAGTTTCAACAC
LeRBOH	ZP2946	CACAAGGTCTAGTTGGAGAACT
	ZP2947	CCATAGTAATTGGAGTCAAGCTC
LeRBOH1	ZP2942	GCTAAAGAACTCAGCCAACT
	ZP4135	AGATGTGTGTACCGTTGATGC
LeWIF1	ZP4133	GGCACCGGCACTAACGAA
	ZP4134	CAACTTAGTGTCCGTGGCAG
NbACTIN	ZP3097	ATGGAAACATTGTGCTCAGTG
	ZP2999	GGTGCTGAGAGAAGCCAAG
NbRBOHA	ZP4136	CTTGCTCGTCAACATCGTG
	ZP4137	GGAGAAATCTTGTTGAGAGC
NbRBOHB	ZP4138	TAATACAAGGAGGGCATATT
	ZP4139	GCAGAACGAGCATCACCT
NbRBOHC	ZP3075	GTGATGACGTGGAAGATCCC
	ZP4145	CCAGTAGGAATTGGGCGTT
NbRBOHD	ZP3079	CAGCTCATCATGCTCTCAAAG
	ZP4146	ATTCCTTAGATTCCCTATTCAT
NbRBOHE1	ZP3096	TTTGTACTCGGATTAATGATAATAG
	ZP3072	CTTAGCCAAAGCATTGAACC
NbRBOHE2	ZP3098	AATTTGCCGGAAATTCCAGT
	ZP3074	TCGAAGCGCGTCTCAACTTT
NbRBOHH	ZP3077	GCTGAAATAGATAGTGATGTTCC
	ZP4144	CCATTCTTGGGACAACTCC
